# The Sensory Profiles of Flatbreads Made from Sorghum, Cassava, and Cowpea Flour Used as Wheat Flour Alternatives

**DOI:** 10.3390/foods10123095

**Published:** 2021-12-14

**Authors:** Rita Dankwa, Heikki Aisala, Eugenie Kayitesi, Henriette L. de Kock

**Affiliations:** 1 Department of Consumer and Food Sciences, University of Pretoria, Private Bag X20, Hatfield, Pretoria 0028, South Africa; eugenie.kayitesi@up.ac.za (E.K.); riette.dekock@up.ac.za (H.L.d.K.); 2 VTT Technical Research Centre of Finland Ltd., Tietotie 2, 02044 Espoo, Finland; Heikki.Aisala@vtt.fi

**Keywords:** sorghum, cowpea, cassava, flatbread, sensory, composites

## Abstract

Sorghum, cowpea, and cassava are underutilised gluten-free sources of flour that have the potential to be used in bread products in sub-Saharan Africa. Excessive wheat imports affect the economies of countries in sub-Saharan Africa, driving the search for wheat flour alternatives. To extend the use of sorghum, cowpea, and cassava flours toward bread production, it is vital that the sensory properties of these flours are better understood. A trained sensory panel evaluated and described the sensory properties of flatbread models prepared from red non-tannin sorghum, fractions (whole and dehulled) of two cowpea varieties, cassava starch, and designated flour composites. The composites were prepared using cassava starch and sorghum flour at 0%, 35%, and 70%, respectively, with 30% cowpea flour. The addition of sorghum intensified sorghum aroma in flatbread, while cowpea flours contributed a beany flavour. Flatbreads from cassava-cowpea composites had a chewier and rubberier mouthfeel, an intense fermented aroma and flavour, and a sour aftertaste compared to single flours, but were most similar to the wheat flatbread, with a residual beany flavour. Information from this study can guide food product developers toward developing new bread products from sorghum, cassava, and cowpea composite flours, thereby moving Africa towards a more sustainable food system. Further research on the effects of the sensory characteristics on consumer liking of the flatbreads is needed.

## 1. Introduction

Wheat-based flatbreads are very popular across sub-Saharan Africa (SSA). A modern chapatti-style flatbread made from wheat, known as East African chapatti, is widely consumed in Kenya, Uganda, Mozambique, Tanzania, and Burundi [1,2]. In Ghana, Nigeria, and South Africa, shawarma (meat wrapped in a wheat flatbread) is gaining much attention [3]. In SSA, only a few countries can grow wheat due to unfavourable climatic conditions [4]. However, SSA countries that produce wheat are unable to meet domestic demand, which results in heavy reliance on wheat imports [5].

Sorghum, cowpea, and cassava are gluten-free climate-resilient crops that are widely cultivated in SSA [6,7,8]. Cowpea and cassava production in SSA alone accounts for >80% and >60% of the world’s production, respectively [7,9], whereas one-third of the world’s sorghum is produced in Africa [10]. Sorghum and cowpeas are important crops but they are largely underutilised as human food [11,12]. Fortunately, flatbreads need less gluten structure for gas holding compared to pan bread, making the inclusion of wheat free flours an option. Thus, gluten-free flours from these crops would be potential alternatives for wheat flour in flatbreads. Research into using the flours to replace wheat flour in flatbreads might benefit SSA economically. If the flours can replace wheat flour, the demand for local production will increase, thereby reducing the need for wheat flour importation.

Sorghum flour is rich in starch (72.3–75.1%), minerals (1.6–1.7%), and polyphenolic compounds (3–43 mg/100 g) [13,14]. Cowpea flour is also high in proteins (20.3–39.4 g/100 g) [15], lysine (7.3–8.74 g/100 g), and resistant starch (12.65%) [16]. Compositing sorghum, cowpea, and cassava flours for flatbread production has potential to improve functional dough or batter properties and the nutritional quality of the bread compared to using the flours individually. According to Patil et al. 2021 [17], dough made from a sorghum and rice composite flour was less sticky and more rollable compared to sorghum dough due to changes in protein-starch and protein-protein interactions. Likewise, the addition of soy flour to rice flour improved batter consistency and bread quality [18]. However, bread consumers generally prefer bread to taste fairly bland, which also explains the popularity of wheat bread [19]. Therefore, successful utilisation of these flours for flatbread production still largely depends on consumer acceptance of their sensory properties. Research on the sensory profile of flatbreads made from sorghum, cowpea, and cassava composite flours has not been explored and described. Such information is important if flours from these crops are to be used for flatbreads.

Dehulling of cowpea seeds is a common practice in the processing of cowpea flour [20] and may influence the sensory properties of food products where the flour is used. Cowpea seed coat contains major components such as phenolic compounds that contribute to flavour, odour and colour of foods [21,22]. Dehulling the cowpea seed improved the appearance and consumer acceptability of “moin-moin” (steamed cowpea bean cake) made from brown cowpeas [23]. Dehulling reduced denseness and improved the colour of griddled cowpea paste [24]. Griddled cowpea paste from dehulled cowpea was preferred over a whole cowpea version due to a better, lighter colour. Fully dehulled cowpeas resulted in a brighter cream colour, a less intense cowpea odour and less stickiness, increased chewiness and better overall liking of Shô basi (a couscous-like product) compared to the product made from partially dehulled white cowpea [22]. This explains why, for certain food applications, dehulling the cowpea seed may be necessary. The impact of dehulling of cowpea on the sensory properties of flatbread made from the flour has not been described in the scientific literature.

Hot and cold paste viscosity varied among 28 cowpea varieties as a result of differences in inherent physical and chemical properties, which may affect texture and other sensory properties of foods (such as baked goods) where the cowpea flour was used [25]. In addition, differences in the size and shape of starch granules, the amount of protein, fats, and several other factors among cowpea varieties had an effect on water absorption [26]. The differences in the seed coat colour among cowpea varieties also influence the colour of the final product [27]. Cassava starch has considerable applications in the chemical and food industries [28]. Compositing cassava starch, sorghum, and cowpea flours could lead to sensory optimisation for bread making purposes. Sayaslan et al. 2000 [29] found cassava starch to have the least volatiles amongst other cereal and tuber starches (corn, wheat, and potato), making it more bland than the other starches. In addition, cassava starch positively affected the texture of pan bread [30]. These qualities might be an advantage for flatbreads containing cassava starch.

Descriptive sensory evaluation is a method used to describe the sensory profile of food products by using trained human assessors. Here a trained sensory panel described the sensory properties of flatbread-type food models prepared from different flours (sorghum, cowpea, and cassava starch) and specific flour combinations. A secondary objective was to understand how the sorghum flour particle size affects the sensory properties of the flatbread. Moreover, the effects of cowpea variety and milling fraction (with and without seed coat, i.e., dehulled) on the sensory profile of the flatbread were considered. Thus, we explored the potential of the flours to replace wheat flour in flatbread.

## 2. Materials and Methods

### 2.1. Preparation of Single Flour Samples

Red, non-tannin, (King Korn super fine sorghum (mabele) meal (about 10% protein) and Snowflake white bread wheat flour were obtained from a local supermarket. White (Bechuana white variety) and red (Glenda variety) cowpea seeds were sourced from Agrinawar Agricol (Pty) Pretoria, South Africa, and cassava starch (about 0.8% protein, dry weight basis) from DADTCO (Dutch Agricultural Development and Trading Company, Inhambane, Mozambique). The initial moisture contents of the white and the red cowpea seeds were 11% and 12%, respectively. The sorghum meal denoted as FSorg was re-milled using a hammer mill (falling number 3100, Perten Instruments, Huddinge, Sweden) fitted with a 500-µm sieve to obtain a finer flour (XFSorg). Whole cowpea seeds were soaked in batches (250 g) at a ratio of 1:1 *w/v* in deionised water in Ziploc bags, at room temperature (~25 °C) for 14 h. The soaked cowpea seeds were then dehulled manually and dried at 45 °C in a forced air convection oven (UF 450, Memmert GmbH, Schwabach, Germany) for 7 h to achieve a moisture content of 7–8%. Whole and dried dehulled cowpea seeds were milled into flour with the same hammer mill and a 500-µm sieve, vacuum-packed in polythene bags, and stored at −20 °C until analyses.

### 2.2. Particles Size Distribution of Sorghum Flours

The particle size distribution of FSorg and XFSorg was determined in triplicate using a procedure described by [31]. Flour aliquots of 100 g were sifted for 10 min in vibrating sieves with mesh sizes of 500, 250, 212, 180, and 150 µm stacked in descending order. The sieves were cleaned after each analysis with a cleaning brush.

### 2.3. Preparation of Composite Flours

XFSorg was composited at 0%, 35%, and 70%, with 30% whole or dehulled cowpea flour and cassava starch (0%, 35%, 70%) by mixing the required amounts for 20 min using a mixer (Kitchen Aid model 5KPM5, St Joseph, MI, USA). The speed was increased every 3–4 min. Composites with 70% cowpea flour or cassava and sorghum flours were not prepared due to the requirement for a nutritional balance of legume and sorghum or cassava flours to ensure a comparable protein content to wheat flour [32,33]. A 100% cassava bread was not included, because such a product mainly contains starch and, therefore, lacks protein for forming a dough network for a flatbread. Moreover, cassava–sorghum bread was not tested because the rational for the composites was to have a product nutritionally similar to the wheat flatbread. On the other hand, both cassava and sorghum are limited in protein content. Twelve different composite flours were prepared (Table 1). The composited flours were vacuum-packed in polythene bags and stored at −20 °C until it was used to make flatbreads.

### 2.4. Descriptive Sensory Evaluation

Descriptive sensory evaluation is traditionally conducted in a laboratory where many factors are controlled during the assessment of product samples. The 2019 COVID-19 pandemic and lockdown restrictions changed work life. The necessity to avoid spreading the COVID-19 virus, to save lives, forced assessors to conduct descriptive sensory evaluations outside of laboratories, at the trained assessors’ own homes. A protocol was developed to control factors that may cause variations in samples during storage, preparation, and sensory evaluation at the assessor’s home.

#### 2.4.1. Flatbread Model Preparation Procedure

Written instructions on the preparation of the flatbreads and a demonstration video were developed for the panel (Supplementary Materials Video S1). The panel was provided with the equipment needed to make the flatbread models (a Teflon pan coated frying pan, a plastic spatula, an induction stove, a frother, 7.5 and 1.25 mL measuring spoons, a basting brush, a 100 mL graduated measuring cylinder, coded cups, and kitchen paper towels). For the preparation, 20 mL of bottled spring water (aQuellé natural spring water) was measured in a 100 mL beaker and poured into a pre-coded plastic cup “batter cup”. The corresponding coded flour (10 g) was poured into the cup and mixed with a battery-operated hand-held coffee frother for a few seconds until a homogenous mixture was formed. Sunflower oil (1.25 mL) was used to grease a Teflon coated pan by spreading the oil with a basting brush. The pan was placed on an induction stove (ZC-6C1, Snappy Chef Trading Limited, Pretoria, South Africa) set at control P3. The batter was stirred again, and 7.5 mL of batter was poured on the marked circular area of the pan. The batter was cooked for 1 min, turned with a spatula, and the other side was cooked for 1 min.

#### 2.4.2. Recruitment, Screening and Training of the Panel

Twelve (12) panellists, students at the University of Pretoria who had been screened for basic sensory acuity were additionally screened using an online questionnaire to assess their understanding of good sensory evaluation practices, access to the internet at home, and willingness and availability to participate. The trained panel consisted of eight (8) females and four (4) males who were aged 18–45 years. A 2-h long introductory session was conducted to introduce the panel to the flatbread preparation and the nature of the sensory evaluation procedure. The written instructions on flatbread preparation, the video and items for preparing the flatbread (Teflon coated frying pan, a plastic spatula, induction stove, frother, 7.5 mL and 1.25 mL measuring spoons, kitchen paper towels, basting brush, a 100 mL graduated measuring cylinder, coded cups, bottled spring water and sunflower oil) were supplied to the panel. Panellists prepared flatbreads from flours (Table 1) and generated descriptors by identifying terms to describe differences among the samples. A 100% cassava flour sample was included in the training to familiarise the panel with the flavour of cassava because it was used to formulate some of the composite flatbreads. Descriptive terms and scale anchors were defined (Supplementary Materials Table S1). Following the method described by [34,35], a consensus list of 33 descriptors was created through online discussions via Blackboard Collaborate™ in another 1-h long session. Subsequently, food references for the descriptive terms suggested by the panel were prepared, and the panel collected these prior to a 2-h online discussion. Another 2-h session was used to train the panel on how to use the intensity scales and to clarify the reference standards depicting the descriptors of each attribute. Details of training and evaluation activities are described in Table S2.

#### 2.4.3. Evaluation of the Flatbread Samples

The panellists collected flour samples prior to a week’s evaluation and an internet link to the evaluation ballot was emailed to them on the day of evaluation. In total, 15 sessions, 3 sessions per week, were conducted. Samples were evaluated in random balanced order following a Williams design. The panel prepared flatbreads from flours in Table 1 and rated the intensities of the appearance, aroma, in-mouth texture, flavour, and aftertaste attributes on unstructured line scales (0–10), with scale end anchors 0 indicating absence and 10 the highest intensity of the attribute. During evaluations, the panel first looked at the cooked samples to evaluate the appearance. They then sniffed all the flatbread samples after removing the lids that covered the plastic cups and recorded the aroma intensities before tasting. They bit and chewed samples to evaluate the in-mouth texture and then the flavour attributes of the samples. Aftertaste was evaluated after the bread was swallowed. Water was used to cleanse their palate between tasting each flatbread sample. The data were collected using Compusense Cloud (Compusense Inc., Guelph, ON, Canada).

### 2.5. Instrumental Colour Measurement of Flours and Flatbreads

A tristimulus colorimeter (CR-400 Chroma meter, Konica Minolta Sensing, Osaka, Japan) was calibrated with a white tile as described by the manufacturer. The (L*) lightness, (a*) red–green, and (b*) blue–yellow colour values of the 7 single and 12 composite flours (Table 1), and the top surface of their cooked flatbreads were measured at three random spots following a procedure described by [36]. Chroma (C*) and hue (H*) were calculated using a* and b* based on standard equations [37]

H* = arctan (b/a) and C* = √a^2^ + b^2^

### 2.6. Statistical Analysis of Data

All the analyses were conducted in triplicate. One-way ANOVA based on 5% significance level was used to test (1) the effect of remilling the sorghum flour on the distribution of particle size and on the sensory properties of sorghum flatbreads; (2) the effects of sorghum or (3) cassava flour levels; or (4) cowpea type in the flatbread (with 30% cowpea flour) on sensory properties. For cowpea only flatbreads, a two-way ANOVA was performed to determine the main and interaction effects of cowpea variety and milling fraction on the sensory attributes of cowpea flatbread or L*, a*, and b* values of cowpea flours and flatbreads. Significant differences between means were calculated using Tukey’s honestly significant difference (HSD) test. The mean scores for sensory attributes of flatbreads made from composite flours were subjected to principal component analysis (PCA). PCA was applied on data standardised based on a correlation matrix. Factor 1 to 6 scores from the PCA were used for agglomerative hierarchical clustering (AHC) of the flatbreads. For AHC, the pairwise similarity and linkage function were measured using Pearson correlation coefficient and the unweighted pair-group average method, respectively. XLSTAT^®^ software was used for analysing the data.

## 3. Results

### 3.1. Particle Size Distribution of Sorghum Flours and the Effects on the Colour of Sorghum Flour and Sorghum Flatbread

**Figure 1:**
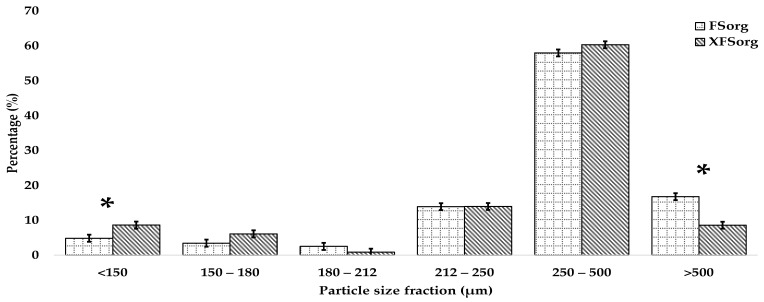
Comparison of particle size (µm) distribution of fine sorghum flour (FSorg) and the fine sorghum flour re-milled with a hammer mill fitted with a 500-µm sieve (XFSorg). The error bars are standard deviations. * Significant differences (*p* < 0.05).

Figure 1 indicates the percentage (%) distribution of different particles size fractions of the sorghum flours. Significant differences were only found between the fractions >500 and <150 µm (*p* < 0.05). The % particles > 500 µm was reduced when sorghum flour was re-milled. Particles between 500 and 250 µm made up the largest portion of the flours. A significant increase from 4.9% to 8.6% (75% increase) in finer particles (<150 µm) was observed after re-milling the sorghum flour.

### 3.2. The Effect of Sorghum Flour Particle Size Profile on the Sensory Properties of Sorghum Flatbread

**Figure 2:**
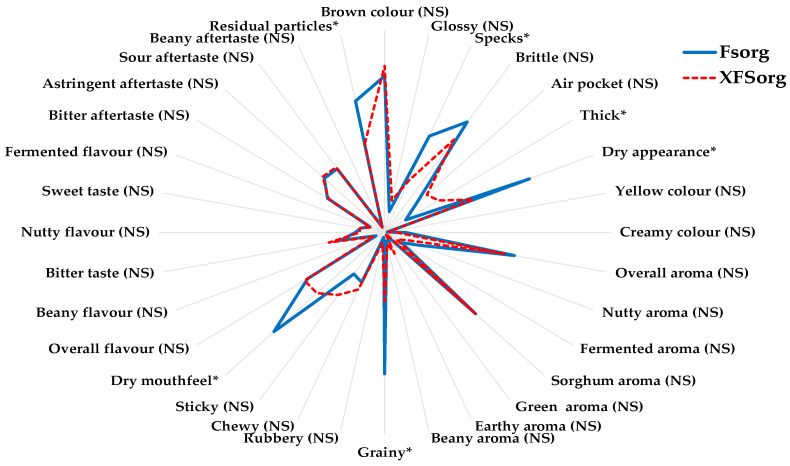
Comparison of sensory profiles of flatbreads made from fine sorghum flour (FSorg) and re-milled fine sorghum flour (XFSorg) as evaluated by a trained panel. * Represents attributes that were significantly different between the sorghum flatbreads (*p* < 0.01); NS = not significant. Each attribute was rated on a scale from 0–10. For interpretation of sensory attributes and reference scales, please refer to Table S1.

Figure 2 shows that the FSorg and XFSorg flatbreads did not differ significantly in aroma and flavour/taste attributes, but there were difference in appearance and in-mouth texture attributes. Residual particles, specks, dry appearance, dry and grainy mouthfeel, and thickness of flatbreads were the sensory attributes that significantly discriminated between the sorghum flatbreads (*p* < 0.05). Flatbread prepared from FSorg with larger particles (>500 µm) had more residual particles, specks, a drier appearance and a grainier mouthfeel than XFSorg flatbread.

### 3.3. The Effect of Sorghum Flour Particle Size Profile on the Colour Parameters of Sorghum Flour and Flatbread

Table 2 shows that re-milling FSorg significantly increased the L* value while decreasing a*, b*, and C* values (*p* < 0.05). The intensity of colour, C* was higher for FSorg than XFSorg flour and flatbread. Even though the XFSorg flour looked lighter (higher L value) than FSorg flour, when batters from the flours were baked into flatbreads, the XFSorg flatbread was darker than FSorg (*p* < 0.001—see also Table 1). The H* value for the sorghum flours and flatbread were lower than 90°, which represents the red and yellow hue quadrant.

### 3.4. Effects of Cowpea Variety and Milling Fraction on Cowpea Flour Colour Properties and Sensory Properties of Cowpea Flatbreads

The L* value of flour from the red cowpea variety (RC) was significantly lower (darker) than flour from the white cowpea variety (WC) (Table 2). Moreover, the dehulled cowpeas flours were lighter (higher L* values) compared to their whole cowpea forms. The whole red cowpea flour (WRC) had the lowest L* value while the dehulled white cowpea flour was the lightest (highest L* value, Table 2). The WC variety and dehulled cowpea flatbreads showed lower a* values compared to the RC variety and whole cowpea flatbreads (Table 2). The C*, on the other hand, did not differ between the white and red cowpea varieties. However, flatbreads made from whole cowpea flour had a significantly lower colour intensity compared to dehulled cowpea flatbread (Table 2). The full details of the main effects of cowpea variety and milling fractions as well as the interaction of the two effects on the mean values for sensory attributes are supplied as Supplementary Materials (Table S3).

**Figure 3:**
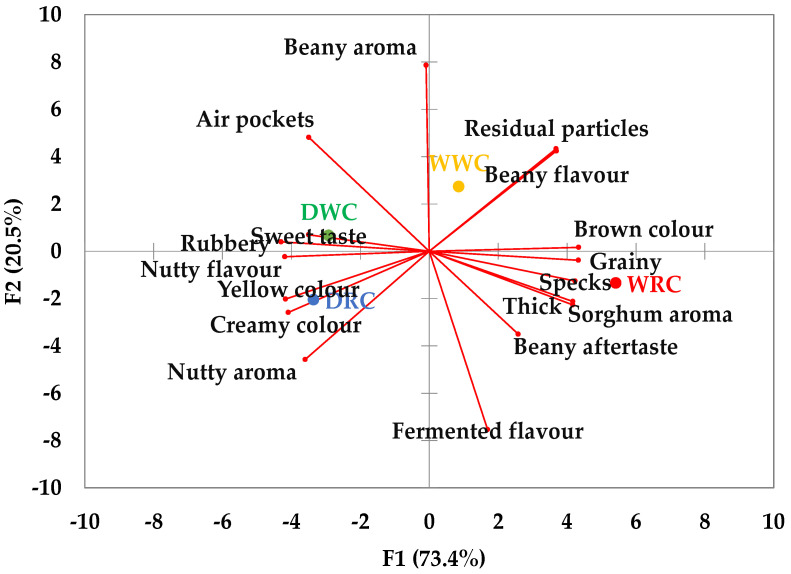
Principal component analysis biplot comparing the sensory attributes of cowpea flatbread models prepared from whole and dehulled flours of a red and a white cowpea variety. WWC = whole white cowpea, WRC = whole red cowpeas, DWC = dehulled white cowpeas, DRC = dehulled red cowpeas. Please note that cowpea flatbreads were evaluated together with sorghum and composite flatbreads by the trained sensory panel.

Figure 3 shows a PCA biplot projecting sensory attribute loadings and product scores of flatbreads on the first two principal factors, which explained 94% of the variation. The first principal factor (F1) separates whole cowpea flatbreads (WWC, WRC) on the right side of the plot from dehulled cowpea flatbreads (DWC, DRC) on the left side of the plot based on colour, residual particles, sorghum aroma, rubberiness, graininess, and thickness. Dehulling the cowpeas resulted in flatbreads that were creamier and yellow coloured, which tasted nuttier and tasted sweeter. In addition, dehulled cowpea flatbreads were rubberier than the whole cowpea flatbreads whereas whole cowpeas flatbreads (WWC, WRC) were darker, thicker, and had a more intense sorghum aroma and more residual grainy particles. The whole cowpea flatbreads were also characterised by a beany aftertaste, visual specks, and a grainy mouthfeel. The second principal factor (F2) explained the variation between the flatbreads made from white (WWC and DWC), at the top of the PCA plot, and the red cowpea variety (WRC and DRC) at the bottom of the plot. The white cowpea flatbread tends to be more “beany”, with a less fermented aroma. However, the difference in the beany aroma, flavour, and aftertaste between the dehulled cowpea flatbreads compared to the whole cowpea flatbreads was not significant (Supplementary Materials, Table S3).

### 3.5. Colour of Flatbreads Prepared from Composite Flours

Table 3 presents mean values for colour parameters of flatbreads made from composite flours. When 70% sorghum flour was composited with 30% cowpea flour, the L* was the smallest for sorghum–whole red cowpea (XFSorg-WRC) whereas cassava starch-dehulled cowpea flatbreads had the highest L* with low redness. Generally, addition of dehulled cowpea flour in the three composite groups (Table 3, sorghum-cowpea, sorghum-cassava-cowpea and cassava-cowpea) increased the L value of flatbreads compared to their whole forms (dehulled > whole white cowpea > whole red cowpea). CS-DRC, CS-DWC, and control had the lowest a* values and were not different in hue angle.

**Figure 4:**
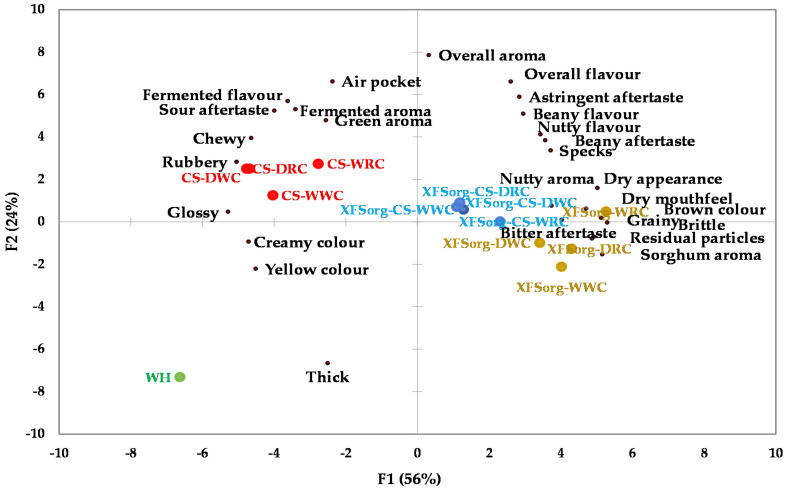
PCA biplot showing sensory characteristics of flatbreads made from sorghum, cowpea, and cassava starch composite flours evaluated by a trained panel (*n* = 12). The abbreviations indicated in red = cassava-cowpea composite flatbreads; blue = sorghum-cassava-cowpea composite flatbreads; brown = sorghum-cowpea composite flatbreads. For flatbread names for the sample abbreviations, please refer to Table 1.

The cassava-cowpea flatbreads were characterised by fermented aroma, fermented flavour, and sour aftertaste and had greener aroma compared to wheat flatbread and flatbreads with sorghum (Figure 4). Fermented flavour was more pronounced with no sorghum (0%) in the composite. Green aroma decreased with the addition of sorghum flour irrespective of the amount added. The flatbreads additionally differed in chewiness, rubberiness, grainy mouthfeel, dry mouthfeel, and beany flavour *p* < 0.05). Flatbreads with 0% sorghum was chewier, rubberier, and less grainy than those with 35% and 70% sorghum. The 70% sorghum flatbreads were less chewy with a drier mouthfeel compared to the 35% sorghum flatbread.

**Figure 5:**
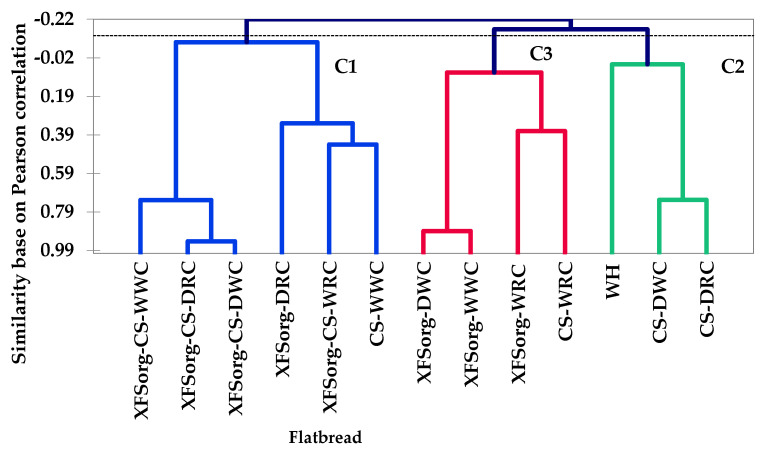
Agglomerative hierarchical clustering (AHC) dendrogram of flatbread models made from composite flours and control by unweighted pair-group average method using the first six factor scores from PCA. The dash line shows position where flatbreads were partitioned into three groups and distinguished by colours. For names of flatbreads, please refer to Table 1.

Figure 5 shows the AHC dendrogram of flatbreads and their relative similarity to each other and the control wheat flatbread based on sensory attributes. The *y*-axis on the dendrogram represents the Pearson correlation coefficient and measures the similarity of the flatbreads. Variance components were 11% for cluster 1, 36% for 2, and 23% for 3, based on ANOVA.

Except for residual particles, C1 flatbreads had similar flavour/taste, aftertaste characteristics, and were comparable in thickness, dry appearance, and specks. Additionally, the C1 flatbreads had similar overall aroma strength compared to C2 and C3. The cassava-cowpea flatbreads were more separated in factor 3, 4, and 5 of the PCA, which was the basis of their separation into different clusters in Figure 5 (Supplementary Materials Table S5). The C2 flatbreads had the least dry mouthfeel, grainy mouthfeel, residual particles, beany flavour, and highest glossiness compared to the other clusters. They were also comparable in colour, sorghum aroma, beany aroma, bitter taste, and aftertaste. Figure 5 shows that the cassava-dehulled cowpea flatbreads were most similar to the wheat flatbread.

## 4. Discussion

### 4.1. How Does Sorghum Flour Particle Size Affect the Sensory Properties of the Flatbread?

The sorghum flatbread prepared with the finer flour (XFSorg) had less specks than FSorg. This is because finer particles are less visible to the eye than larger particles. The higher L* and lower a* of XFSorg flour compared to FSorg was because it had a greater amount of finer particles with a larger surface area to increase light reflection. This was in agreement with earlier reports by [38,39], where L* showed a significant increase with decreasing particle size and finer flour appeared brighter. Interestingly, when batters from the flours were baked, FSorg flatbread was the driest and appeared whiter than the XFSorg flatbread. Instrumental measurement revealed that XFSorg flatbread had the highest colour intensity (C*).

The fineness of the flour also influenced other aspects of the bread surface appearance and thickness of the flatbread. Finer milling of sorghum flour contributed positively to the texture perception of the flatbread. It reduced the perception of grainy or gritty particles and dry mouthfeel, resulting from the smaller ratio of particles larger than 500 µm. Graininess or grittiness of sorghum products are perceived more readily with increased flour particle size and the amount of larger particles [40]. Flatbread from FSorg felt grainier during eating due to a greater percentage of coarse particles (>500 µm) compared to XFSorg. During eating or chewing, saliva is released to lubricate the oral surfaces [41] and larger particles increase friction between oral surfaces, reducing food hydration with saliva potentially contributing to a dry mouthfeel. According to [42,43] finer flour particles compared to coarse flour particles have higher starch damage and a larger surface area exposed to water in doughs or batters which increases their ability to bind and hold water. Thus, the batter made from FSorg probably absorbed and bound less water allowing the unbound water to evaporate during baking. Additionally, more damaged starch in XFSorg flour will swell faster compared to FSorg in the presence of heat and leach out amylose and amylopectin more quickly. This causes an increase in batter viscosity [44] and greater flatbread thickness. Notably, the character and intensity of aroma and flavour were similar for the flatbreads prepared with FSorg and XFSorg flours.

### 4.2. How Do Cowpea Variety and Milling Fraction (with and without Seed Coat, i.e., Dehulled) Affect the Sensory Profile of the Flatbread?

As expected, photographs showed that the whole cowpea flatbreads were brown whereas the dehulled cowpea flatbreads were yellow–green. The white cowpea flatbread appeared brighter and less red (a*) than flatbread from the red cowpea variety. The difference in the colour of the cowpea varieties is due to the different seed coat colour. The red cowpea flatbread was browner due to more anthocyanins concentrated in the seed coat of the red seeds [27].

The red cowpea flatbread smelled less beany compared to the white one. Beany aroma or beany flavour is a limiting factor in the acceptance of bread [45] and many other foods [46,47]. Differences in phenolic compounds in the cowpeas could account for the differences in the beany aroma of the flatbreads from the red and the white varieties. During harvesting of the cowpea seeds, lipoxygenase catalyses the oxidation of unsaturated fatty acids, resulting in undesirable beany aroma in legumes [48]. The more pigmented red cowpeas have higher total phenolic compounds [49] that offer higher resistance to oxidation of fats, possibly explaining the lesser beany aroma in flatbread made from red cowpeas. Phenolic compounds inhibit lipoxygenase activity by binding to lipoxygenase to prevent complexing with unsaturated fatty acids and abstracting hydrogens from unsaturated fatty acid.

Flatbreads from dehulled cowpea flours were nuttier and sweeter compared to whole cowpea flour flatbread. Flatbread with nutty aroma may be desirable. Micronised and boiled cowpeas with nutty flavours were preferred by consumers [36,50]. Nuttier aroma and flavour for dehulled compared to whole boiled cowpeas was also reported by [50]. Nutty aroma and flavour in legumes are reported to be caused by formation and release of flavour compounds such as pyrazines during Maillard reaction as a result of heat application [51,52]. Tannins are more concentrated in cowpea seed coat [53] and the removal of the seed coat may have increased the perception of nutty aroma and flavour. In whole cowpeas, during baking, tannins in the seed coat may form complexes with proteins [54,55], thus reducing the amount of free amino acids available to react with reducing sugars during Maillard reaction. The sweeter taste of flatbread from dehulled cowpea flour agrees with results reported for boiled dehulled cowpeas [56]. The researchers attributed the sweetness to a relative increase in reducing sugars after dehulling. Pal et al. 2017 [57] reported more total soluble sugars in dehulled versus whole lentil flour. Cowpea seed coats contain fibre and polyphenols that may dilute or mask the sweet taste in whole flour cowpea flatbread.

Dehulled cowpea flatbreads appeared less thick than whole cowpea flatbreads. This could be attributed to the reduction in fibre content due to dehulling. A study by [58] found that dehulling reduced the soluble fibre content in cowpea. Lower total soluble fibre in dehulled cowpea flour deceases the ability of the flour to bind and entrap water compared to the whole cowpea flour. Thus, more entrapped water in the whole cowpea flatbread caused the flatbread to be thicker. A rubberier mouthfeel in the dehulled cowpea flour flatbreads compared to the whole cowpea flour flatbreads was probably due to the change in the starch to fibre ratio. As the fibre proportion decreases, a rubberier mouthfeel could result. Irrespective of the cowpea variety, whole cowpea flour flatbreads were grainier leaving more residual particles in the mouth due to the presence of cowpea seed coat residues in whole cowpea flours.

### 4.3. Sensory Properties of Flatbread-Type Food Models Prepared from Different Flours (Sorghum, Cowpea, and Cassava Starch) and Specific Flour Combinations

The sorghum-cowpea flatbreads were browner compared to flatbreads with cassava flour added. The flatbreads with a higher proportion of sorghum were browner due to the contribution of pigments in the sorghum pericarp [13,59]. Cassava starch in the flatbread contributed a creamier, slightly yellow colour after baking. The colour of cassava-cowpea flatbread was more similar to the wheat flatbread. The differences in the colour of the flatbreads may not be undesirable because consumers appreciate brown and white bread products on the market.

The lower brittleness in sorghum-cowpea flatbread compared to the sorghum flatbreads (Fsorg and XFSorg) may be due to an increase in protein content from cowpea [60]. The sorghum flour had protein content of 10% while protein content of the sorghum-cowpea flours ranged from 15–17% (not presented here). The globulins, the main storage protein in cowpea are hydrophilic unlike kafirin (the main storage protein in sorghum) and will have greater affinity for water molecules to increase water absorption and binding capacity increasing retention of moisture in sorghum-cowpea flatbreads [59]. However, the high proportion of sorghum in sorghum-cowpea flatbreads, still contributed to the moderately dry appearance of sorghum-cowpea flatbreads and grainy residues in the mouth. Sorghum endosperm contains floury and horny portions. The horny endosperm are difficult to mill in relation to the floury endosperm and coarse particles result from the milling of the horny endosperm sorghum [44]. In addition, starch granules remained encapsulated by the hydrophobic kafirin [61] restricting the starch granules’ ability to absorb water. Similar findings of a dry appearance was reported for sorghum-cowpea biscuit samples [62]. When the proportion of sorghum was reduced to 35% and substituted by 35% cassava starch, the dry appearance of the flatbread decreased as the cassava substitution increased the total starch content. Cassava starch contained only a small amount of non-starch components and had a higher ability to retain moisture due to its high swelling power [63]. The sorghum-cassava-cowpea and cassava-cowpea flatbreads were chewier and rubberier probably due to a high starch content. Starch contributes to viscosity, texture and mouthfeel [64]. Cassava starch has a low gelatinisation temperature (about 60 °C [65]. The low gelatinisation temperature of cassava starch compared to sorghum flour [66] means cassava starch gelatinises more rapidly and more gelatinised starch may have increased the batter viscosity. Higher cohesion [67] contributed to rubberiness and chewiness of the flatbreads. With 70% cassava starch, the rubberiness and chewiness was more pronounced, making cassava starch-cowpea flatbreads rubberier and chewier than wheat flatbread.

The aftertaste of cowpea flatbreads was more astringent than sorghum flatbreads. Single flours from cowpea may not be suitable for flatbread due to the intense beany aroma, flavour, and aftertaste. Likewise, the high brittleness, dry appearance, bitter aftertaste and dry mouthfeel make sorghum flour alone unsuitable for flatbread. The astringent aftertaste of the composited flour flatbreads did not differ probably because the cowpea proportion in the flatbreads was kept constant.

While cowpea variety affected the beany aroma intensity in cowpea flour only flatbreads, there was no difference in beany aroma when the two cowpea varieties were composited with sorghum and cassava starch. This may be due to the lower proportion of cowpea flour in the flatbreads made from composite flours compared to flatbreads from only cowpea flours.

The nutty aroma in the flatbreads was mainly contributed by sorghum and cowpea; it could have formed during baking by the Maillard reaction. Differences in the nutty aroma of flatbreads can be attributed to differences in protein content of the composites as a result of the flour combinations. The cassava starch has a small amount of protein (0.9%) and therefore cassava-dehulled cowpea flatbreads (8–9% protein in composite flour) were lower in nutty aroma compared to sorghum-cowpea and sorghum-cassava-cowpea (9–13% protein in composite flours). Nuttiness in cassava-cowpea flatbreads was similar to wheat flatbread.

The cassava starch in the composites introduced fermented aroma and flavour in the flatbreads which were more pronounced in cassava-cowpea flatbreads. The fermented aroma and flavour were probably caused by volatile and non-volatile organic acids in cassava starch that may have been as a result of the wet extraction method for production of the starch. Wet extraction of cassava starch involves milling cassava with water, filtration, separating sediment from filtrate to obtain the wet starch and drying over a 24-h period at a low temperature [68]. The separation of the starch from the suspension is reported to be a critical step that should be completed in as short a time as possible [69]. This is because the suspension contains sugars that could be fermented by microorganisms producing organic acids [69]. In spite of this, fermented flavour may not be an undesirable sensory attribute for bread consumers because fermented flavour is a desirable attribute of many traditional African foods [62,70]. The cassava-dehulled cowpea flatbreads were perceived to be the most similar to wheat flatbread but had a more beany and fermented flavour. Pre-treating the cowpea by roasting slightly before grinding and a blander cassava starch might be an option to obtain a more bland composite flour for bread manufacture.

## 5. Conclusions

This study documents the sensory properties of sorghum, cowpea, cassava, and their composited flours when applied in a model flatbread type product. Flatbreads made from a composite of 70% cassava starch and 30% dehulled cowpea flour were most similar to the wheat flour flatbread, but with a residual beany flavour. Single flours from sorghum, cowpea, and cassava are less suitable for the manufacture of flatbread. Sorghum flour particle size affected some appearance, mouthfeel, and after swallowing characteristics. Finely milled sorghum flour will be an option to minimise dry appearance, graininess, and residual particles in flatbreads. The beany flavour differences between cowpea varieties observed in the 100% cowpea flatbreads were not apparent in the composites. Therefore, composites can be made from either cowpea variety. Dehulling cowpeas affected colour, in-mouth texture, taste, and residual particles. Dehulling cowpeas before compositing the flour with cassava starch is desirable to simulate wheat flatbread flour. Cassava starch is good for inclusion in composites flours owing to its functionality to contribute rubbery and chewy texture. Follow-up research is needed to upscale the formulations to commercial scale flatbread baking and to determine consumer acceptance. This study provides valuable information that food product developers could use to develop bread products using these climate-resilient gluten-free flours to increase their utilisation and move SSA countries towards a more sustainable food system.

## Figures and Tables

**Table 1:** Description of single flours, composite flours, and images of flatbread prepared from the flours.

	Flours	(%)	Abbreviations	Images of the Flatbreads
Single flours				
Wheat	Wheat	100	WH	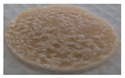
Sorghum	Fine sorghum flour	100	FSorg	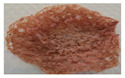
	Extra fine sorghum fine	100	XFSorg	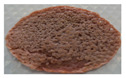
Cowpea	Whole red cowpea	100	WRC	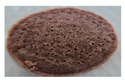
	Whole white cowpea	100	WWC	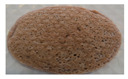
	Dehulled red cowpea	100	DRC	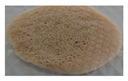
	Dehulled white cowpea	100	DWC	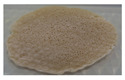
Composite flours				
Sorghum-cowpea	Sorghum/whole red cowpea	70:30	XFSorg-WRC	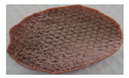
	Sorghum/whole white cowpea	70:30	XFSorg-WWC	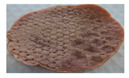
	Sorghum/dehulled red cowpea	70:30	XFSorg-DRC	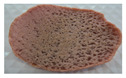
	Sorghum/dehulled white cowpea	70:30	XFSorg-DWC	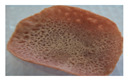
Sorghum-cassava-cowpea	Sorghum/cassava/whole red cowpea	35:35:30	XFSorg-CS-WRC	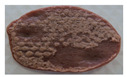
	Sorghum/cassava/whole white cowpea	35:35:30	XFSorg-CS-WWC	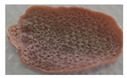
	Sorghum/cassava/dehulled red cowpea	35:35:30	XFSorg-CS-DRC	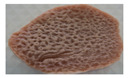
	Sorghum/cassava/dehulled white cowpea	35:35:30	XFSorg-CS-DWC	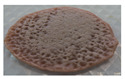
Cassava-cowpea	Cassava/whole red cowpea	70:30	CS-WRC	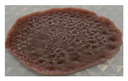
	Cassava/whole white cowpea	70:30	CS-WWC	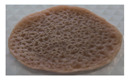
	Cassava/dehulled red cowpea	70:30	CS-DRC	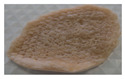
	Cassava/dehulled white cowpea	70:30	CS-DWC	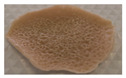

**Table 2:** The effect of remilling of sorghum flour and the effects of cowpea variety and milling fraction and the interaction of these factors on instrumental colour parameters of flours and flatbreads.

	Colour Parameter									
	Flours					Flatbreads				
	L*	a*	b*	C*	H*	L*	a*	b*	C*	H*
**Effect of remilling sorghum flour**										
Fine sorghum FSorg	79.8 (0.1)	4.6 (0.1)	11.9 (0.1)	12.8 (0.1)	68.8 (0.3)	50.9 (0.3)	10.0 (0.3)	13.4 (0.2)	16.8 (0.0)	53.2 (1.2)
Extra fine sorghum XFSorg	82.4 (0.0)	4.4 (0.0)	10.9 (0.0)	11.7 (0.0)	68.2 (0.1)	43.6 (0.5)	6.7 (0.3)	8.6 (0.2)	10.9 (0.3)	52.1 (1.0)
p-value	**	*	*	**	NS	***	*	**	**	NS
**Cowpea variety**										
Red cowpea RCTTKKWhite cowpea WC	87.3 (0.1)	1.0 (0.0)	10.6 (0.1)	10.6 (0.1)	84.2 (0.2)	59.0 (0.2)	4.47 (0.1)	17.5 (0.3)	18.5 (0.3)	72.9 (0.5)
	89.8 (0.0)	0.6 (0.0)	9.7 (0.1)	9.7 (0.1)	86.2 (0.1)	61.2 (0.4)	2.7 (0.2)	17.5 (0.4)	17.8 (0.4)	79.2 (0.6)
p-value	***	***	***	***	***	**	***	NS	NS	***
**Cowpea milling fraction**										
Whole cowpea	85.4 (0.1)	1.5 (0.0)	8.9 (0.1)	9.1 (0.1)	80.8 (0.2)	52.9 (0.3)	5.7 (0.1)	12.4 (0.2)	13.7 (0.1)	65.6 (0.7)
Dehulled cowpea	91.6 (0.0)	0.1 (0.0)	11.3 (0.0)	11.3 (0.0)	89.6 (0.0)	67.3 (0.2)	1.4 (0.2)	22.5 (0.5)	22.6 (0.5)	86.5 (0.4)
p-value	***	***	***	***	***	***	***	***	***	***
**Interaction of cowpea variety and milling fraction**										
Whole red WRC	84.3 (0.3) ^a^	1.8 (0.1) ^d^	9.4 (0.1) ^a^	9.6 (0.1) ^a^	79.2 (0.3) ^a^	51.3 (0.2)	7.3 (0.1) ^a^	12.6 (0.3) ^a^	14.6 (0.2) ^b^	59.9 (0.9) ^a^
Whole white WWC	86.5 (0.0) ^a^	1.1 (0.0) ^c^	8.5 (0.2) ^a^	8.6 (0.2) ^a^	82.5 (0.1) ^b^	54.4 (0.7) ^a^	4.1 (0.2) ^b^	12.2 (0.2) ^a^	12.9 (0.1) ^a^	71.4 (1.2) ^b^
Dehulled red DRC	90.2 (0.1) ^a^	0.2 (0.0) ^b^	11.7 (0.0) ^a^	11.7 (0.0) ^a^	89.2 (0.0) ^c^	66.9 (0.3) ^a^	1.6 (0.2) ^a^	22.3 (0.6) ^b^	22.4 (0.6) ^c^	85.8 (0.4) ^c^
Dehulled white DWC	93.0 (0.1) ^a^	0.0 (0.0) ^a^	10.9 (0.0) ^a^	10.9 (0.0) ^a^	90.0 (0.0) ^c^	68.0 (0.3) ^a^	1.2 (0.3) ^a^	22.7 (0.8) ^b^	22.7 (0.8) ^c^	87.1 (0.6) ^c^
p-value	NS	***	NS	NS	***	NS	***	**	**	*

Values in parenthesis represent standard errors. * *p* < 0.05, ** *p* < 0.01, *** *p* < 0.001, NS = Not significant. L* = 100 for lightness, and 0 for darkness; a* = chromaticity from green (−) to red (+); b* = chromaticity from blue (−) to yellow (+); H* = hue angle; C* = Chroma. Values with different superscript letters within a column for a section differed significantly (*p* < 0.05).

**Table 3:** Mean values (±standard deviations) for colour parameters for flatbreads made from composite flours and control.

			Colour Parameters		
Flatbread	L*	a*	b*	C*	H*
Control wheat	58.8 (0.4) ^ef^	0.4(0.1) ^a^	15.3(0.0) ^f^	15.3 (0.0) ^ef^	88.5 (0.2) ^g^
Sorghum-cowpea					
XFSorg-WRC	51.3 (3.5) ^bc^	8.1 (0.7) ^i^	18.2 (0.9) ^g^	19.9 (1.0) ^g^	66.0 (2.0) ^d^
XFSorg-WWC	56.7 (0.5) ^e^	5.0 (0.1) ^f^	11.2 (0.4) ^ab^	12.3 (0.4) ^abc^	65.8 (0.6) ^d^
XFSorg-DRC	52.5 (0.4) ^c^	7.0 (0.4) ^h^	13.1 (0.4) ^de^	14.8 (0.5) ^def^	61.9 (0.7) ^c^
XFSorg-DWC	62.2 (0.6) ^fg^	4.8 (0.1) ^e^	12.9 (0.8) ^cde^	13.7 (0.8) ^cde^	69.6 (0.8) ^e^
Sorghum-cassava-cowpea					
XFSorg-CS-WRC	47.1 (0.3) ^a^	8.0 (0.1) ^i^	10.8 (0.5) ^a^	13.4 (0.4) ^bcd^	53.4 (1.5) ^a^
XFSorg-CS-WWC	52.9 (0.3) ^c^	5.4 (0.1) ^g^	10.5 (0.4) ^a^	11.8 (0.4) ^ab^	62.8 (1.4) ^cd^
XFSorg-CS-DRC	53.3 (0.5) ^cd^	6.5 (0.2) ^h^	14.2 (0.3) ^ef^	15.8 (0.2) ^f^	65.5 (1.0) ^d^
XFSorg-CS-DWC	56.3 (1.6) ^de^	4.4 (0.2) ^d^	11.7 (0.2) ^abcd^	12.5 (0.2) ^abc^	69.6 (0.6) ^e^
Cassava-cowpea					
CS-WRC	47.9 (0.1) ^ab^	7.6 (1.0) ^hi^	11.5 (0.8) ^abc^	13.7 (1.2) ^cde^	57.0 (1.9) ^b^
CS-WWC	57.0 (0.4) ^e^	2.8 (0.2) ^c^	11.1 (0.3) ^a^	11.6 (0.3) ^a^	75.73 (0.8) ^f^
CS-DRC	62.4 (0.7) ^g^	1.0 (0.1) ^b^	12.7 (0.2) ^bcd^	12.7 (0.2) ^abc^	85.5 (0.3) ^g^
CS-DWC	62.7 (0.3) ^g^	0.6 (0.1) ^a^	12.9 (0.5) ^cde^	12.9 (0.5) ^abc^	87.3 (0.6) ^g^

Values are the means of three replicates. Means compared with Tukey’s (HSD) test. Means within a column denoted by different superscripts differ significantly (*p* < 0.05). XFSorg = extra fine sorghum flour, WWC = whole white cowpea, WRC = whole red cowpea, DWC = dehulled white cowpea, DRC = dehulled red cowpea, CS = cassava starch. L* = 100 for lightness, and 0 for darkness; a* = chromaticity from green (−) to red (+); b* = chromaticity from blue (−) to yellow (+); H* = hue angle; C* = Chroma.

## Data Availability

The data presented in this study are available upon request from the authors.

## Supplementary Materials

The following are available online at https://www.mdpi.com/article/10.3390/foods10123095/s1, Table S1: Sensory descriptors and food reference guide used by the trained descriptive sensory panel; Table S2: Training schedule for descriptive sensory evaluation of the flatbread samples; Table S3: Sensory attributes of cowpea flatbread from different varieties and milling fractions; Table S4: Effect of different composite ratios of cassava starch and sorghum flours on the sensory attributes of flatbreads; Table S5: First six factor scores of principal component analysis of 33 attributes of flatbreads from composites flours and control wheat flour, Video S1; Video demonstration on flatbread preparation.
