# Supplementary material for: The Sensory Profiles of Flatbreads Made from Sorghum, Cassava, and Cowpea Flour Used as Wheat Flour Alternatives
Source: Foods. 2021 Dec 14;10(12):3095. doi: 10.3390/foods10123095 (PMC8701489; doi:10.3390/foods10123095)
Supplement: Supplementary file 1 [file foods-10-03095-s001.zip › Table S1.pdf]

Table S1. Sensory descriptor lexicon used by the trained descriptive sensory panel to evaluate the flatbreads.

Descriptors	Definitions	Reference	Rating scale
**Appearance**			**(0-10)**
Brown	The degree to which the sample appears brown on the surface	Golden brown sugar = 3<br>Chocolate milk drink = 10	Not brown<br>Very brown
Creamy colour	The degree to which the sample appears cream on the surface	Whey concentrate powder = 1<br>Almond butter biscuit (Daisy Health Foods) = 5	Not creamy<br>Very creamy
Yellow	The degree to which the sample appears creamy on the surface	very yellow (like sunflower oil) =10	Not yellow<br>Very yellow
Spongy	The visual assessment of the surface texture of the sample	Very dense, no air cells = 0<br>Very spongy (resembling a sponge)<br>Sponge = 10	Not spongy<br>Very spongy
Glossy	The degree to which the surface of the flatbread appears glossy	Not glossy (or matt, no reflection) to very glossy (like egg white)	Not glossy<br>Very glossy
Specks	The degree to which visible specks are present on the surface of the sample	No specks to many specks (almost covering the top surface)	No specks<br>Many specks
Brittle	The degree to which the sample appears delicate and fragile	Not brittle (flexible and soft) to very brittle (like potato chips)	Not brittle<br>Very brittle
Air pocket	The degree to which pores are visible on the surface ranging from no pores to many pores (almost covering the top surface)	No air pocket = 0<br>Many air pocket = 10	No air pocket<br>Many air pockets
Thick	The thickness of the sample	Thin and flat to very thick (more than 5mm)	Not thick<br>Very thick
Dry	The degree to which one side of the flatbread appears dry and powdery	Not dry (looks very moist and wet) to very dry (a dried layer on the surface)	Not dry<br>Very dry
**Aroma**			
Intensity of overall aroma	The intensity of the aroma of the sample		No aroma<br>Intense aroma

Descriptors	Definitions	Reference	Rating scale
Earthy aroma	The intensity of the aroma associated with damp black soil		No earthy aroma<br>Intense earthy aroma
Beany aroma	The intensity of the aroma associated with bean and bean food products	Cooked sugar beans = 6	No beany aroma<br>Intense beany aroma
Sorghum aroma	Intensity of aroma associated with sorghum	Cooked sorghum porridge = 5	No sorghum aroma<br>Intense sorghum aroma
Green aroma	The intensity of green aroma of fresh cut grass or cooked green vegetables	Cooked spinach=10	No green aroma<br>Intense green aroma
Nutty aroma	The intensity of the aroma associated with roasted nuts	Roasted Peanuts = 5<br>Peanut butter =10	No nutty aroma<br>Intense nutty aroma
Fermented aroma	The intensity of the aroma associated with a fermented product like sauerkraut	Sauerkraut = 10	No fermented aroma<br>Intense fermented aroma
In-mouth texture/Mouthfeel			
Grainy	The degree to which the sample feels grainy in the mouth as a result of the presence of small particles	Cooked coarsely milled sorghum porridge = 10	Not grainy<br>Very grainy
Rubbery	The degree to which the sample feels rubbery during chewing similar to chewing a wine gum sweet	Woolworths wine gum = 10	Not rubbery<br>Very rubbery
Chewy	The degree to which the sample does not disintegrate when chewed (needs several chews required before swallowing)	Needs 10 or more chews = 10	Not chewy<br>Very chewy
Dry	The degree to which the sample feels dry while chewing	Oatcakes biscuit (Nairn's fine milled Oatcakes) = 10 very dry (absorbs saliva)	Not dry<br>Very dry
Sticky	The degree to which the sample feels sticky and sticks to the teeth and palate while eating		Not sticky<br>Very sticky
Flavour/Taste			
Overall flavour	The intensity of the perceived flavour		Not intense<br>Very intense
Bitter taste	The intensity of the bitter taste associated with caffeine perceived.	0.15 % caffeine in water = 10	Not bitter<br>Very bitter

Beany flavour	The intensity of the flavour associated with bean and bean products	Cooked sugar bean (boiled for 2 hours) = 6	Not beany<br>Very beany
Nutty flavour	The inensity of the nutty flavour characterised by the presence of roasted nuts	Roasted Peanuts = 5<br>Peanut butter = 10	Not nutty<br>Very nutty
Sweet taste	The intensity of a basic sweet taste associated with sugar	2% sugar in water=10	Not sweet<br>Very sweet
Fermented flavour	The intensity of the flavour associated with fermented products	Sauerkraut = 10	Not fermented<br>Very fermented
Aftertaste	After swallowing		
Bitter aftertaste	The intensity of the bitter taste associated with caffeine perceived after swallowing the sample.	0.15 % Caffeine in water = 10	Not bitter<br>Very bitter
Astringent	The intensity of a dry sensation associated with strong black tea	Black tea (2 bags soaked in 750 ml hot boiled water) = 7	Not astringent<br>Very astringent
Sour aftertaste	The intensity of a sour taste of which citric acid is typical	0.08% Citric acid=10	Not sour<br>Very sour
Beany aftertaste	The intensity of a beany flavour after swallowing the sample	Cooked sugar bean (boiled for 2 h) = 6	Not beany<br>Very beany
Residual particles	The degree to which particles are perceived in the mouth after swallowing the sample		No particles<br>Many particles
