# Supplementary material for: The Sensory Profiles of Flatbreads Made from Sorghum, Cassava, and Cowpea Flour Used as Wheat Flour Alternatives
Source: Foods. 2021 Dec 14;10(12):3095. doi: 10.3390/foods10123095 (PMC8701489; doi:10.3390/foods10123095)
Supplement: Supplementary file 1 [file foods-10-03095-s001.zip › Table S2.pdf]

Table S2. Training schedule for descriptive sensory evaluation of the flatbread samples.

Session	Hour	Objective	Activity
Introduction	2	To introduce the nature of the sensory evaluation procedure and flatbread preparation method	1. The sensory evaluation method (descriptive sensory evaluation was explained to the panelists.<br>2. Panelists were shown how to prepare flatbread for evaluation.<br>3. Explain how to use the equipment.<br>4. The panelist received written instructions, a copy of a video on flatbread preparation and equipment after this session.
Training	1	To generate descriptors for the flatbreads	5. The panel were presented with eight different single flours (FSorg, XFSorg, WRC, DRC, WWC DWC ,WH and cassava starch) to prepare flatbread following the standard method provided to them.
	1		6. They evaluated and generated descriptors for the flatbreads.
	1		7. Online discussions among panelists to agree on descriptors that were identified.
	2	To generate the final list of descriptors and familiarized with attributes defined	8. The list of attributes was adjusted through discussion to obtain a final list.<br>9. Terms used to discriminate flatbreads were defined and scale anchors and references also developed for descriptors.
	2	To introduce scaling for the descriptors	10. Panelist received references for anchoring scale of each descriptor.<br>11. Consensus discussion on the references to anchor the scales<br>12. Panelists were trained to use the intensity scale.
	2	To familiarize with the evaluation process	13. The panel evaluated flatbread samples in a test situation.<br>14. Four flour samples (WWC, DRC, CS-DWC and XFSorg) coded with three-digit numbers collected.<br>15. The flour samples were prepared into flatbreads and evaluated in duplicate.<br>16. Also, DWC, XFSorg-WWC, XFSorg-CS-DWC were prepared and evaluated as in step 13-14
	2		
Final evaluation	2 (15 sessions)		17. Nineteen flour samples and cassava starch (not reported here) were prepared as flatbreads and evaluated in triplicate during 15 sessions.<br>18. Panelist received three sets of flour samples to prepared flatbreads for the three evaluation days per week. Each sample set (comprised of four flour samples assigned a three-digit code), batter and evaluation cups were date-coded for each evaluation day.<br>Panelists prepared and uploaded photos of the prepared flatbread samples prior to evaluation.

*Please refer to Table 1 for the description of the flatbread samples
