# Supplementary material for: The Sensory Profiles of Flatbreads Made from Sorghum, Cassava, and Cowpea Flour Used as Wheat Flour Alternatives
Source: Foods. 2021 Dec 14;10(12):3095. doi: 10.3390/foods10123095 (PMC8701489; doi:10.3390/foods10123095)
Supplement: Supplementary file 1 [file foods-10-03095-s001.zip › Table S3.pdf]

Table S3. Sensory attributes of cowpea flatbread from different varieties and milling fractions.

Sensory attribute	Cowpea variety		p-value	Milling fraction		p-value	Interaction effect				p-value
	RC	WC		Whole	Dehulled		WWC	WRC	DWC	DRC	
**Appearance**											
Brown colour	4.8^{b}	2.7^{a}	**< 0.0001**	7.0^{b}	0.4^{a}	**< 0.0001**	4.8^{b}	9.3^{c}	0.6^{a}	0.3^{a}	**< 0.0001**
Creamy colour	4.2^{a}	4.7^{a}	0.267	1.5^{a}	7.4^{b}	**< 0.0001**	2.4^{b}	0.7^{a}	7.1^{c}	7.7^{c}	**0.019**
Yellow colour	1.7^{a}	1.7^{a}	0.92	0.4^{a}	3.0^{b}	**< 0.0001**	0.7^{a}	0.2^{a}	2.8^{a}	3.2^{a}	0.242
Spongy	5.7^{a}	5.5^{a}	0.716	5.2^{a}	5.9^{a}	0.195	4.9^{a}	5.5^{a}	6.0^{a}	5.8^{a}	0.467
Glossy	1.2^{a}	0.9^{a}	0.323	1.0^{a}	1.1^{a}	0.698	0.7^{a}	1.3^{a}	1.1^{a}	1.1^{a}	0.414
Specks	2.5^{a}	2.0^{a}	0.436	2.7^{a}	1.8^{a}	0.115	2.2^{a}	3.3^{a}	1.9^{a}	1.7^{a}	0.289
Brittle	0.9^{a}	0.8^{a}	0.852	0.8^{a}	0.9^{a}	0.628	0.8^{a}	0.8^{a}	0.9^{a}	1.0^{a}	0.794
Air Pocket	5.8^{a}	6.7^{a}	0.065	6.0^{a}	6.6^{a}	0.225	6.6^{a}	5.4^{a}	6.8^{a}	6.3^{a}	0.452
Thick	7.1^{a}	6.3^{a}	0.118	7.2^{b}	6.2^{a}	**0.027**	6.6^{a}	7.9^{a}	6.1^{a}	6.3^{a}	0.206
Dry	3.1^{a}	3.3^{a}	0.814	3.3^{a}	3.1^{a}	0.814	3.4^{a}	3.1^{a}	3.1^{a}	3.2^{a}	0.638
**Aroma**											
Overall aroma	7.0^{a}	6.8^{a}	0.639	6.9^{a}	6.9^{a}	0.876	6.9^{a}	6.9^{a}	6.7^{a}	7.0^{a}	0.639
Earthy aroma	2.2^{a}	2.0^{a}	0.727	2.3^{a}	2.2^{a}	0.562	2.1^{a}	2.4^{a}	1.9^{a}	2.0^{a}	0.908
Sorghum aroma	1.6^{b}	0.8^{a}	**0.018**	1.8^{b}	0.6^{a}	**0.000**	1.0^{a}	2.6^{b}	0.6^{a}	0.6^{a}	**0.022**
Beany aroma	1.8^{a}	2.9^{b}	**0.013**	2.5^{a}	2.2^{a}	0.498	3.1^{a}	2.0^{a}	2.8^{a}	1.6^{a}	0.837
Green aroma	1.3^{a}	1.9^{a}	0.196	1.5^{a}	1.7^{a}	0.649	1.8^{a}	1.2^{a}	1.9^{a}	1.4^{a}	0.916
Nutty aroma	3.7^{a}	3.3^{a}	0.489	2.8^{a}	4.2^{b}	**0.014**	2.8^{a}	2.9^{a}	3.9^{a}	4.5^{a}	0.626
Fermented aroma	1.2^{a}	1.2^{a}	0.897	1.1^{a}	1.3^{a}	0.652	1.0^{a}	1.2^{a}	1.4^{a}	1.1^{a}	0.606
**In-mouth texture/Mouthfeel**											

Grainy	2.2^{a}	1.7^{a}	0.354	2.5^{b}	1.4^{a}	**0.014**	2.0^{a}	3.0^{a}	1.4^{a}	1.3^{a}	0.244
Rubbery	1.4^{a}	1.8^{a}	0.279	1.0^{a}	2.1^{b}	**0.009**	1.4^{a}	0.7^{a}	2.2^{a}	2.1^{a}	0.457
Chewy	5.9^{a}	6.0^{a}	0.842	5.8^{a}	6.1^{a}	0.465	5.6^{a}	6.0^{a}	6.4^{a}	5.9^{a}	0.289
Sticky	2.3^{a}	2.2^{a}	0.822	2.2^{a}	2.3^{a}	0.955	2.1^{a}	2.3^{a}	2.2^{a}	2.3^{a}	0.911
Dry	3.7^{a}	3.4^{a}	0.602	3.8^{a}	3.2^{a}	0.238	3.7^{a}	3.9^{a}	3.1^{a}	3.4^{a}	0.934
**Flavour/Taste**											
Overall flavour	5.8^{a}	5.7^{a}	0.838	5.8^{a}	5.7^{a}	0.838	5.8^{a}	5.9^{a}	5.7^{a}	5.8^{a}	1.00
Beany flavour	2.8^{a}	3.0^{a}	0.65	3.3^{a}	2.5^{a}	0.086	3.3^{a}	3.3^{a}	2.7^{a}	2.3^{a}	0.65
Bitter taste	1.8^{a}	1.7^{a}	0.736	1.8^{a}	1.7^{a}	0.783	1.8^{a}	1.8^{a}	1.5^{a}	1.9^{a}	0.691
Nutty flavour	3.3^{a}	3.5^{a}	0.697	3.0^{a}	3.9^{a}	0.086	3.4^{a}	2.6^{a}	3.6^{a}	4.1^{a}	0.222
Sweet taste	0.9^{a}	1.3^{a}	0.143	0.8^{a}	1.4^{b}	**0.011**	0.9^{a}	0.7^{a}	1.7^{a}	1.2^{a}	0.48
Fermented flavour	1.6^{a}	1.0^{a}	0.172	1.4^{a}	1.3^{a}	0.975	1.0^{a}	1.7^{a}	1.1^{a}	1.5^{a}	0.726
**Aftertaste**											
Bitter aftertaste	1.8^{a}	1.3^{a}	0.206	1.6^{a}	1.5^{a}	0.784	1.4^{a}	1.8^{a}	1.1^{a}	1.9^{a}	0.68
Astringent aftertaste	4.0^{a}	4.3^{a}	0.609	4.1^{a}	4.3^{a}	0.784	4.3^{a}	3.9^{a}	4.4^{a}	4.1^{a}	0.919
Sour aftertaste	1.6^{a}	1.6^{a}	0.963	1.3^{a}	1.8^{a}	0.253	1.6^{a}	1.0^{a}	1.5^{a}	2.1^{a}	0.182
Beany aftertaste	2.5^{a}	2.2^{a}	0.459	2.4^{a}	2.3^{a}	0.702	1.9^{a}	3.0^{a}	2.5^{a}	2.0^{a}	0.063
Residual particles	2.9^{a}	3.2^{a}	0.52	3.8^{b}	2.3^{a}	**0.001**	3.8^{a}	3.7^{a}	2.5^{a}	2.0^{a}	0.703

p-values in bold are significant at $p<0.05$. Means within a row denoted by different superscripts differ significantly ($p<0.05$). RC= Red cowpea; WC= White cowpea; WWC= Whole white cowpea; WRC= Whole red cowpea; DWC= Dehulled white cowpea; DRC =Dehulled red cowpea.
