# Supplementary material for: The Sensory Profiles of Flatbreads Made from Sorghum, Cassava, and Cowpea Flour Used as Wheat Flour Alternatives
Source: Foods. 2021 Dec 14;10(12):3095. doi: 10.3390/foods10123095 (PMC8701489; doi:10.3390/foods10123095)
Supplement: Supplementary file 1 [file foods-10-03095-s001.zip › Table S4.pdf]

Table S4. Effect of different composite ratios of cassava starch and sorghum flours on the sensory attributes of flatbreads.

Sensory attribute	0%Cassava starch (70 % Sorghum)	35% Cassava starch (35 % Sorghum)	70% Cassava starch (0 % sorghum)	p-value
Appearance				
Brown colour	7.6(0.2)[c]	6.1(0.2)[b]	2.5(0.3)[a]	<.0001
Creamy colour	1.1(0.2)[a]	1.9(0.2)[b]	5.5(0.3)[c]	<0.0001
Yellow colour	0.3(0.1)[a]	0.3(0.1)[a]	2.5(0.3)[b]	<0.0001
Spongy	2.3(0.3)[a]	0.3(0.3))[a]	2.6(0.3)[a]	0.411
Glossy	1.1(0.2)[c]	2.9(0.3)[b]	4.3(0.3)[a]	<0.0001
Specks	2.3(0.2)[a]	2.3(0.2)[a]	1.8(0.3)[a]	0.235
Brittle	3.2(0.3)[b]	2.2(0.3))[a]	1.4(0.2)[a]	<0.0001
Air Pocket	2.4(0.2)[a]	3.0(0.3)[a]	4.6(0.3)[b]	<0.0001
Thick	3.9(0.2)[a]	3.7(0.2)[a]	3.9(0.2)[a]	0.767
Dry	2.6(0.2)[a]	3.3(0.3)[a]	2.6(0.2)[a]	<0.0001
Aroma				
Overall aroma	6.2(0.2)[a]	6.5(0.2)[ab]	7.0(0.2)[b]	0.005
Earthy aroma	1.8(0.2)[a]	1.9(0.2)[a]	1.6(0.2)[a]	0.734
Sorghum aroma	3.4(0.2)[c]	2.6(0.2)[b]	0.8(0.2)[a]	<0.0001
Beany aroma	1.4(0.2)[a]	1.6(0.2)[a]	2.1(0.2)[a]	0.062
Green aroma	1.0(0.2)[a]	1.2(0.2)[a]	2.4(0.7)[b]	<0.0001
Nutty aroma	2.2(0.2)[b]	2.1(0.2)[b]	1.4(0.2)[a]	0.014
Fermented aroma	0.9(0.1)[a]	1.7(0.2)[b]	4.1(0.3)[c]	<0.0001
In-mouth texture/Mouthfeel				
Grainy	2.8(0.3)[c]	1.9(0.2)[b]	0.8(0.2)[a]	<0.0001
Rubbery	0.9(0.1)[a]	2.7(0.2)[b]	6.6(0.2)[c]	<0.0001
Chewy	4.4(0.2)[a]	5.9(0.2)[b]	7.7(0.2)[c]	<0.0001
Sticky	3.3(0.3)[a]	3.1(0.2)[a]	2.9(0.3)[a]	0.513
Dry	4.6(0.23)[c]	3.0(0.2)[b]	1.6(0.2)[a]	<0.0001
Flavour				
Overall flavour	4.4(0.2)[a]	4.8(0.2)[a]	4.4(0.2)[a]	0.23
Beany flavour	1.8(0.2)[ab]	2.3(0.2)[b]	1.6(0.2)[a]	0.038
Bitter taste	2.1(0.2)[a]	1.9(0.2)[a]	1.6(0.2)[a]	0.222
Nutty flavour	1.5(0.2)[a]	2.1(0.2)[a]	1.5(0.2)[a]	0.107
Sweet taste	1.0(0.1)[a]	1.1(0.1)[a]	1.2(0.1)[a]	0.51
Fermented flavour	0.7(0.2)[a]	1.3(0.2)[a]	2.6(0.3)[b]	<0.0001
After swallowing				
Bitter	2.4(0.2)[b]	1.9(0.2)[ab]	1.3(0.2)[a]	0.001
Astringent	3.5(0.3)[a]	3.3(0.3)[a]	3.3(0.3)[a]	0.779
Sour	1.0(0.2)[a]	1.1(0.2)[a]	1.9(0.2)[b]	0.004
Beany	1.2(0.2)[a]	1.8(0.2)[a]	1.2(0.2)[a]	0.093
Residual particles	3.7(0.3)[c]	2.5(0.2)[b]	1.7(0.2)[a]	<0.0001

Means within a row denoted by different superscripts differ significantly ($p<0.05$). Values in parenthesis represent standard errors.
