# Supplementary material for: The Sensory Profiles of Flatbreads Made from Sorghum, Cassava, and Cowpea Flour Used as Wheat Flour Alternatives
Source: Foods. 2021 Dec 14;10(12):3095. doi: 10.3390/foods10123095 (PMC8701489; doi:10.3390/foods10123095)
Supplement: Supplementary file 1 [file foods-10-03095-s001.zip › Table S5.pdf]

Table S5. First six factor scores of principal component analysis of 33 attributes of flatbreads made from composites flours and wheat flour.

Flatbreads	F1	F2	F3	F4	F5	F6
Wheat	-6.625	-7.315	-0.249	0.380	0.540	0.035
XFSorg-WRC	5.257	0.486	-0.793	2.397	1.003	0.610
XFSorg-WWC	4.024	2.118	-0.253	0.634	-1.393	-0.922
XFSorg-DRC	4.311	1.269	0.809	1.716	0.729	1.133
XFSorg-DWC	3.415	-0.988	-0.252	0.311	-1.577	-0.285
XFSorg-CS-WRC	2.306	0.015	-1.038	-0.451	1.332	0.787
XFSorg-CS-WWC	1.281	0.580	0.342	-0.767	0.114	-0.241
XFSorg-CS-DRC	1.096	0.706	1.407	-0.987	1.515	-1.228
XFSorg-CS-DWC	1.184	0.910	1.475	-0.086	0.704	-0.348
CS-WRC	-2.773	2.732	-3.526	-0.451	-0.223	-0.535
CS-WWC	-4.039	1.250	-0.565	-1.351	-0.054	0.441
CS-DRC	-4.763	2.504	1.480	0.481	-1.389	0.412
CS-DWC	-4.674	2.507	1.163	1.607	0.155	0.142

F1, F2, F3, F4, F5 and F6 are the first-six principal factors used for the dendrogram. Please refer to Table 1 for the names of the flatbreads.
