# Supplementary material for: The Sensory Profiles of Flatbreads Made from Sorghum, Cassava, and Cowpea Flour Used as Wheat Flour Alternatives
Source: Foods. 2021 Dec 14;10(12):3095. doi: 10.3390/foods10123095 (PMC8701489; doi:10.3390/foods10123095)
Supplement: Supplementary file 1 [file foods-10-03095-s001.zip › Video S1.pdf]

Video S1. Video demonstration on flatbread preparation.
https://zenodo.org/record/5564403#.YXGZihpByMo
